# Supplementary figures and images for: Binding to DCAF1 distinguishes TASOR and SAMHD1 degradation by HIV-2 Vpx
Source: PLoS Pathog. 2021 Oct 26;17(10):e1009609. doi: 10.1371/journal.ppat.1009609 (PMC8570500; doi:10.1371/journal.ppat.1009609)

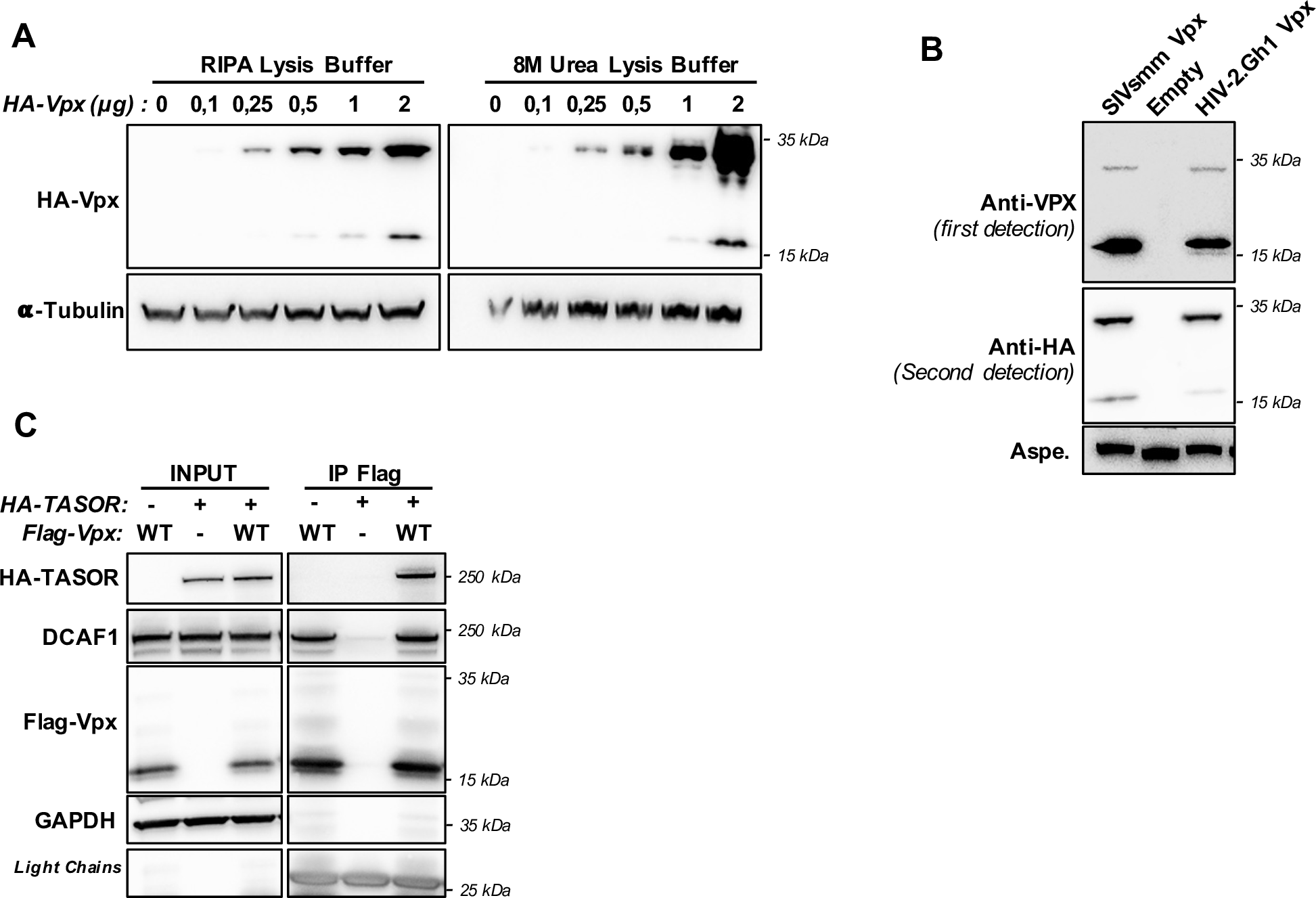

Supplement: S1 Fig — (A) The 30-kDa form of Vpx is non-denaturable in RIPA or 8M Urea lysis buffer. Increasing quantities of plasmid encoding HA-Vpx WT were expressed in HeLa cells and then the cells were lysed by RIPA lysis buffer or 8M Urea lysis buffer (composition: 8M Urea; 2M Thiourea; 4% CHAPS; 30mM Tris HCl pH 8,5). (B) The 30-kDa form of Vpx is revealed by an anti-Vpx antibody. HA-Vpx HIV-2.Gh1 or HA-Vpx SIVsmm were expressed in HeLa cells. The whole cells extract was analyzed by Western-blot, first by using an-anti-Vpx antibody, and then with an anti-HA antibody. (C) Flag-Vpx WT from HIV-2.Gh1 interacts with HA-TASOR. Flag-Vpx WT was co-expressed with HA-TASOR short isoform in HeLa cells, then an anti-Flag immunoprecipitation was performed. (TIF) [file ppat.1009609.s001.tif]

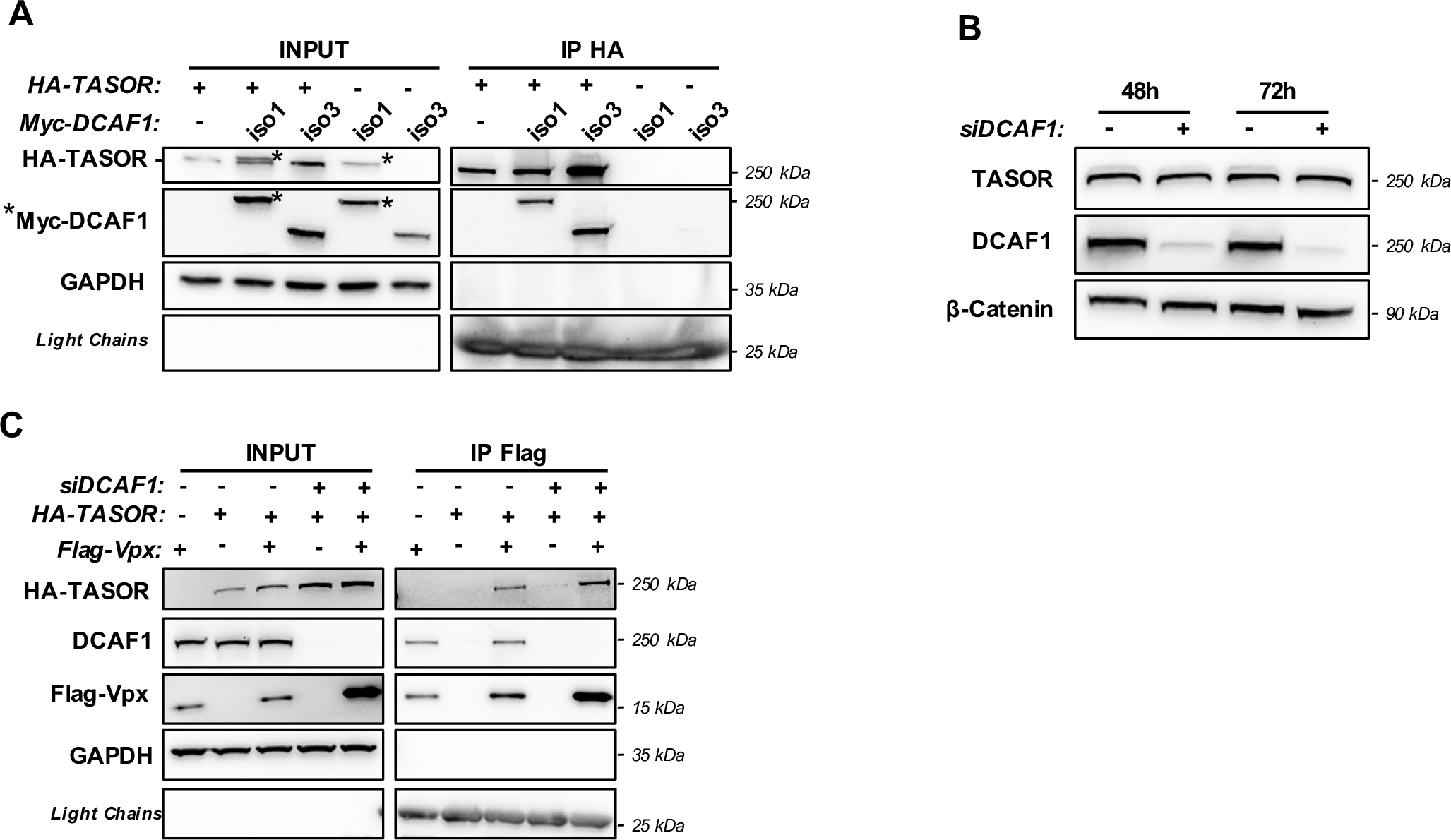

Supplement: S2 Fig — (A) TASOR interacts with the two isoforms of DCAF1 in an overexpression system. HA-TASOR short isoform was co-expressed with Myc-DCAF1 isoform 1 (iso1) or isoform 3 (iso3) in HeLa cells, then an anti-HA immunoprecipitation was performed. (*) The Myc-DCAF1 band is detected in the HA-TASOR panel. (B) Endogenous TASOR protein level is not affected following DCAF1 depletion. HeLa cells were transfected with 40nM of siCTL (-) or siDCAF1 (+) and cells were harvested at 48h and 72h. (C) Flag-Vpx interacts with HA-TASOR in absence of DCAF1. HeLa cells were treated with siRNA CTL or siRNA DCAF1. After 24h, Flag-Vpx WT was co-expressed with HA-TASOR for 48h, then an anti-Flag immunoprecipitation was performed. In each panel, the indicated proteins were revealed by western blot. (TIF) [file ppat.1009609.s002.tif]

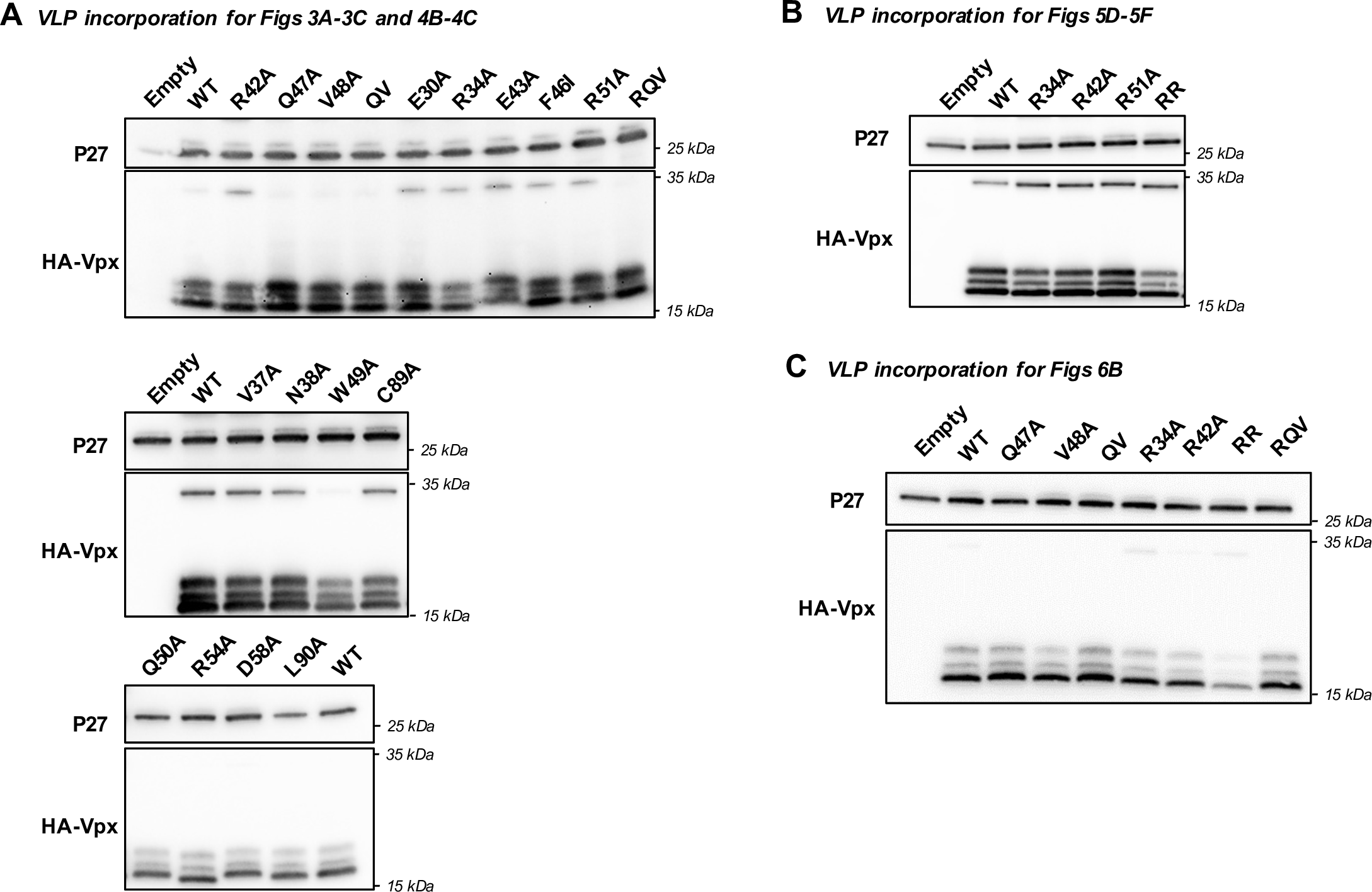

Supplement: S3 Fig — For each panel, VLP were produced in 293FT by co-transfection of a packaging vector, an envelope VSVg vector and a vector encoding HA-Vpx (WT or mutants). 72h post transfection, supernatants were harvested and the VLPs were concentrated by ultracentrifugation. 12 μL of each were analyzed by western blot. VLP production was checked with anti-P27 (HIV-2 capsid) antibody and HA-Vpx incorporation with an anti-HA antibody. (A) Western blot of VLP incorporation for Figs 3A, 3B, 3C, 4B and 4C. (B) Western Blot of VLP incorporation for Fig 5D, 5E and 5F. (C) Western Blot of VLP incorporation for Fig 6B. (TIF) [file ppat.1009609.s003.tif]

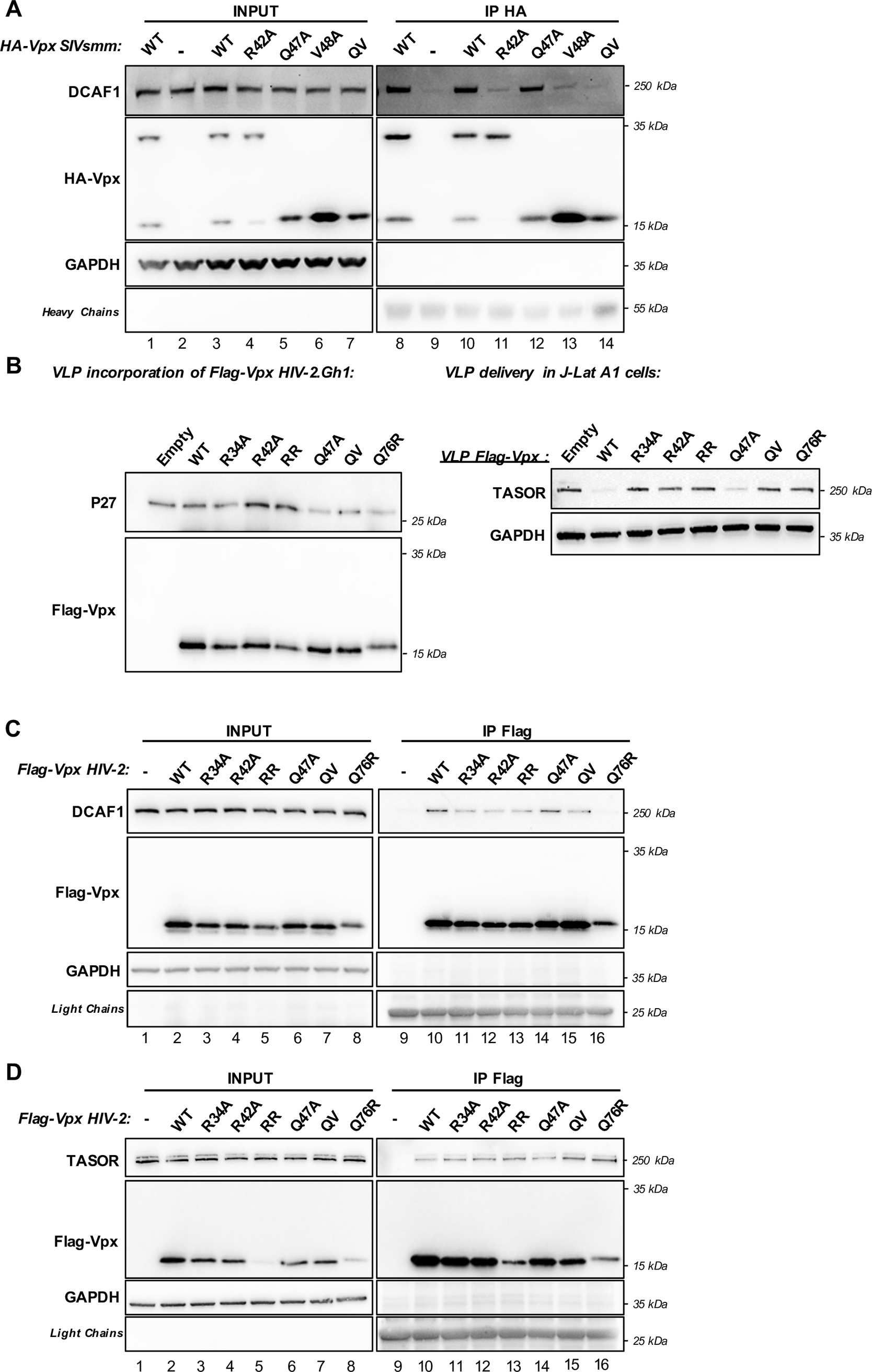

Supplement: S4 Fig — (A) SIVsmm Vpx R42A, V48A and QV mutants show a lower binding affinity to DCAF1. HA-Vpx WT from SIVsmm or indicated mutants were expressed in HeLa cells, then an anti-HA immunoprecipitation was performed. (B) TASOR degradation phenotype of Flag-Vpx HIV-2.Gh1 WT or mutants. Left. Analysis of Flag-Vpx (WT and mutants) incorporation into VLP by western-blot. Right. J-Lat A1 T cells were treated with VLPs containing Flag-Vpx WT or indicated mutants. After overnight treatment, the whole cell extracts were analyzed by western blot. The immunoblot is representative of 3 independent experiments. (C and D) Flag-tagged Vpx R34A, R42A, RR and QV mutants are less affine for DCAF1 (C) but not for TASOR (D). Flag- HIV-2.Gh1 Vpx WT or indicated mutants were expressed in HeLa cells, then an anti-Flag immunoprecipitation was performed. The shown Immunoblots are representative of 2 independent experiments. (TIF) [file ppat.1009609.s004.tif]

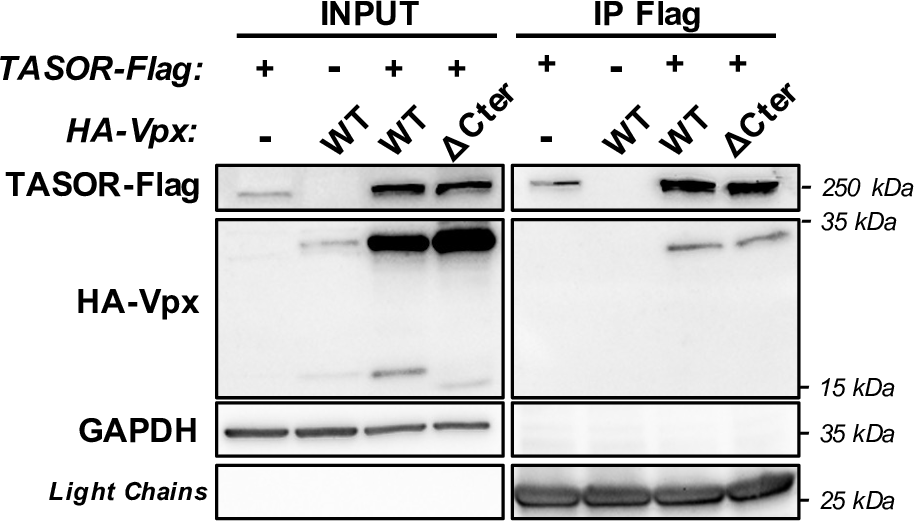

Supplement: S5 Fig — HA-Vpx WT or HA-ΔC-ter Vpx and TASOR-Flag were co-expressed in HeLa cells, then an anti-HA immunoprecipitation was performed. (TIF) [file ppat.1009609.s005.tif]

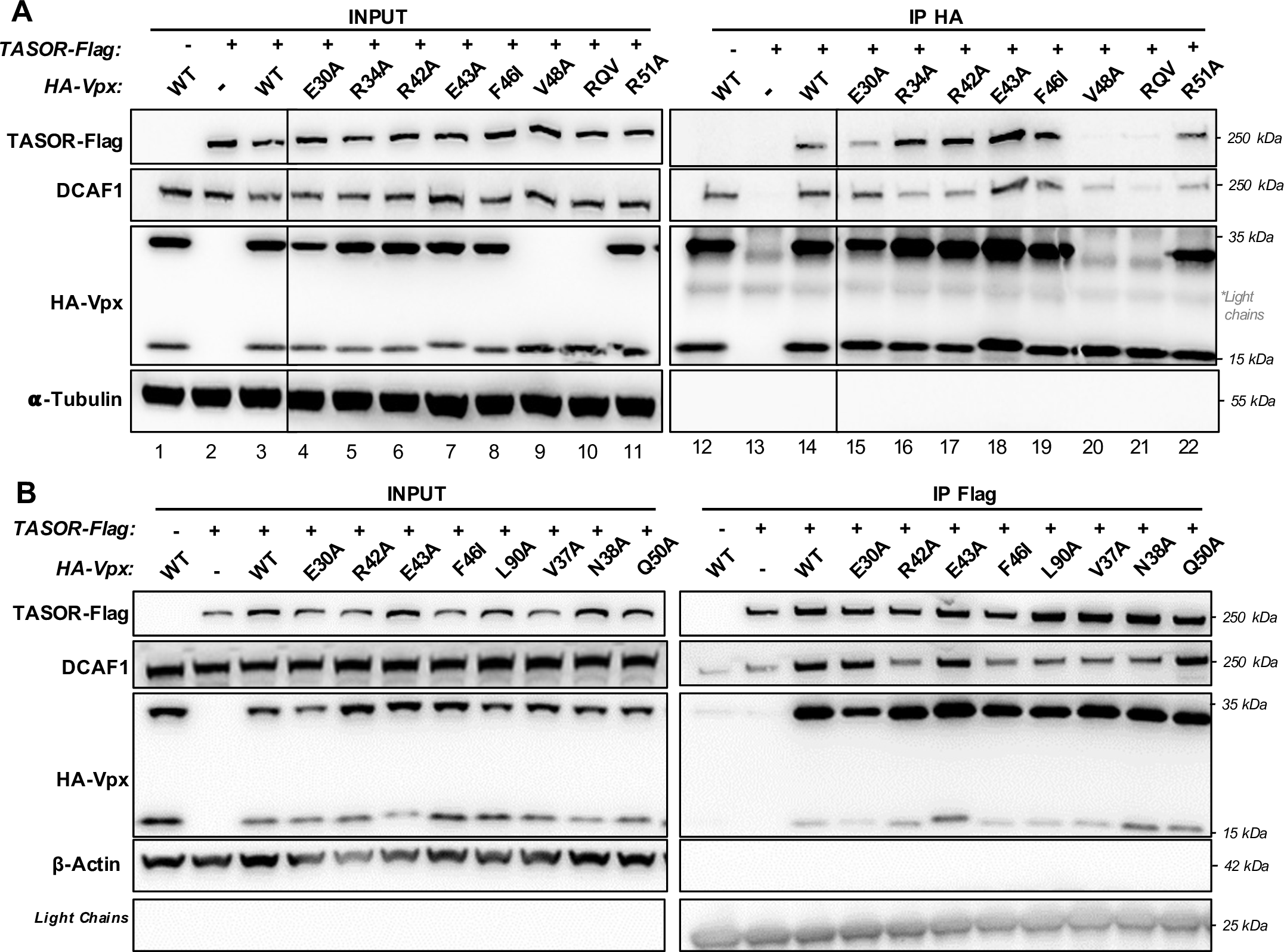

Supplement: S6 Fig — (A) HA-Vpx WT or indicated mutants and TASOR-Flag were co-expressed in HeLa cells, then an anti-HA immunoprecipitation was performed. (B) TASOR-Flag and HA-Vpx WT or indicated mutants were co-expressed in HeLa cells, then an anti-Flag immunoprecipitation was performed. (TIF) [file ppat.1009609.s006.tif]

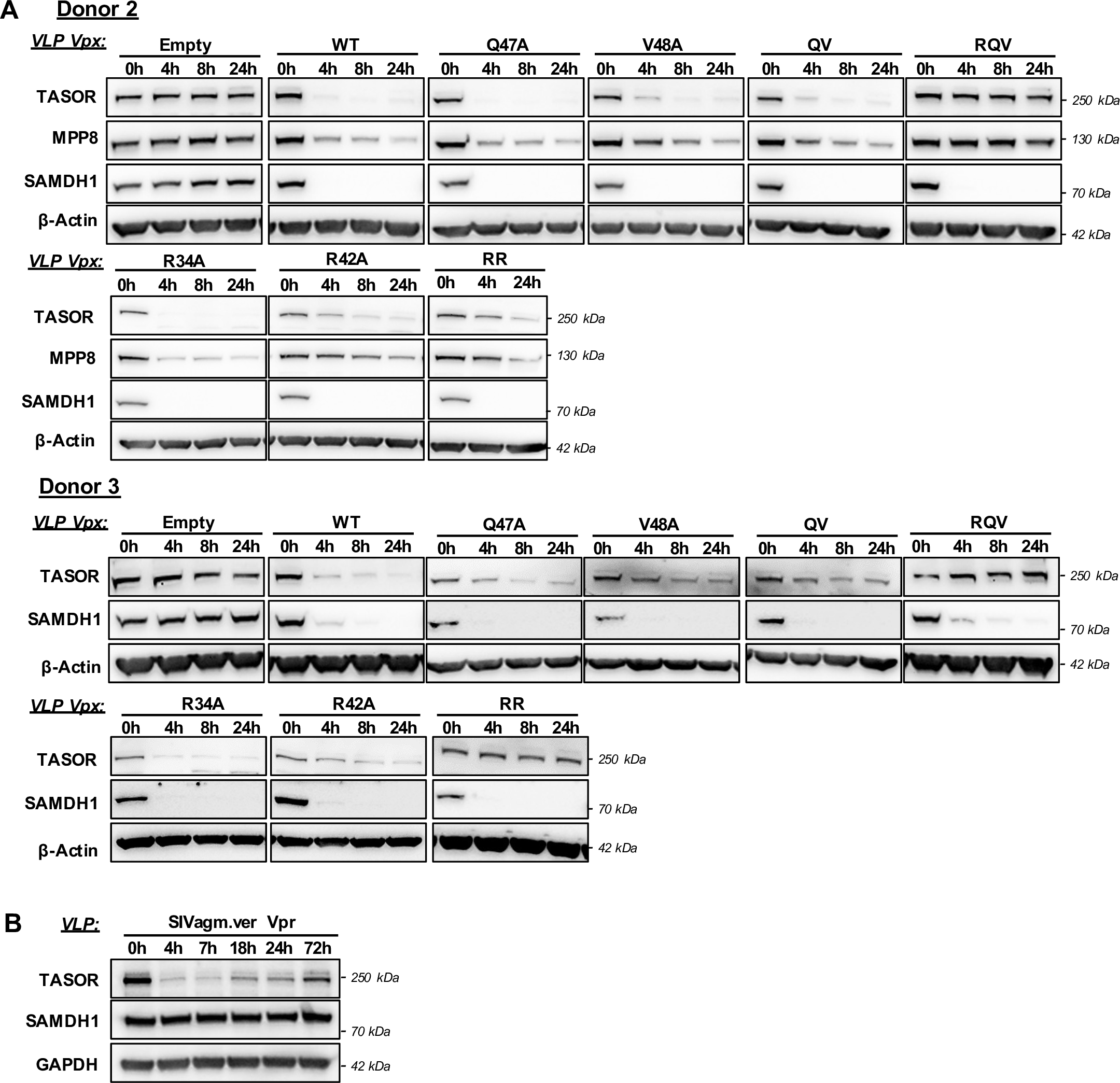

Supplement: S7 Fig — (A) Short Kinetics of TASOR and SAMHD1 degradations by HIV-2.Gh1 Vpx WT or mutants delivered to Monocyte-derived-Macrophages (MDM) by VLP.Purified monocytes from healthy donors were differentiated 7 days with GM-CSF and M-CSF. After differentiation, MDM were transduced with indicated Vpx-containing VLPs and harvested at indicated times. Whole-cell extracts were analyzed by western-blot. QV; Vpx Q47A-V48A double mutant. RQV: Vpx R42A-Q47A-V48A triple mutant. RR: Vpx R34A-R42A double mutant. (B) Short kinetic of TASOR degradation by SIVagm.ver9063 Vpr in MDM. SIVagm.ver Vpr is unable to induce human SAMDH1 degradation. (TIF) [file ppat.1009609.s007.tif]
