# Supplementary material for: Binding to DCAF1 distinguishes TASOR and SAMHD1 degradation by HIV-2 Vpx
Source: PLoS Pathog. 2021 Oct 26;17(10):e1009609. doi: 10.1371/journal.ppat.1009609 (PMC8570500; doi:10.1371/journal.ppat.1009609)
Supplement: Attachment — Submitted filename: Martin_Matkovic_response_reviewers_29092021.docx [file ppat.1009609.s009.docx]

Reviewer's Responses to Questions

Reviewer #1: Within their manuscript “Binding to DCAF1 distinguishes TASOR and SAMHD1 degradation by HIV-2 Vpx”, Martin and colleagues characterize the interaction of HIV-2 Vpx with the component of the HUSH complex TASOR in great detail on a molecular level.
The manuscript is nicely written and the experiments are technically sound and well controlled. The study is of some interest to the field as it adds new molecular details on the nature of the Vpx-mediated degradation of the HUSH complex/TASOR.
Within a series of convincing immunoprecipitation and western blot experiments Martin et al found that TASOR binds to the E3-Ligase adaptor molecule DCAF1, even in the absence of Vpx. However, HIV-2 Vpx binding to TASOR and DCAF1 stabilizes this interaction. In addition, the authors found that the N-terminal domain of TASOR is important for Vpx interaction and were able to identify patches in Vpx crucial for TASOR inactivation and promoting HIV mini genome expression. Interestingly, the authors also found that the residues in Vpx important for binding to TASOR differs from the ones important for SAMHD1 interaction.

We thank the reviewer for their comments.

Reviewer #2: The manuscript by Martin and Matkovic et al. describes a rigorous and thorough structure-function analysis of HIV-2 Vpx interaction with known partners DCAF1, TASOR and SAMHD1. Whereas the determinants of Vpx accessory protein from HIV-2/SIVsm have been quite well studied regarding SAMHD1 antagonism, much remained to be understood in the context of HUSH complex degradation. In this context, the authors did an extensive mutagenesis analysis in order to characterize Vpx interactions with DCAF1, TASOR and SAMHD1. Whereas DCAF1 interaction with SAMHD1 depends on Vpx, the authors report here that DCAF1 was able to interact with TASOR in the absence of Vpx, but the latter did increase/stabilize the interaction. Then the authors showed the importance of the PARP-like domain of TASOR in DCAF1 binding; of note this domain was not important for Vpx binding, suggesting that DCAF1 and Vpx bind to different regions of TASOR. Moreover, the authors interestingly identified a series of Vpx mutants unable to induce TASOR degradation while remaining active against SAMHD1. Surprisingly, some of these mutants had a poor binding affinity for DCAF1 but not for TASOR, which highlighted the essential role of DCAF1 in Vpx-mediated degradation of TASOR. In addition, this raised the question of whether little amounts of DCAF1 could be sufficient for Vpx-mediated degradation of SAMHD1, or if an alternative partner could be used in this context. As expected, there was a strict correlation between the ability of Vpx mutants to degrade TASOR and to enhance HIV-1 minigenome expression in a J-lat A1 T cell model. Finally, the authors globally confirmed their observations in primary monocyte-derived-macrophages. Of note, some of the Vpx mutants described here will be particularly useful to specifically study HUSH restriction in primary myeloid cells, where SAMHD1 is also active. The manuscript is very well written, the data are clear and convincing, the findings are highly interesting and they will certainly be of great interest for people in the field. There are only a few minor points to address.

We thank the reviewer for their comments.

Reviewer #3: The manuscript authored by Martin and coworkers expands on previous observations by the Margottin-Goguet lab relating to the ability of Vpx to degrade the HUSH complex. In this work, Martin et al. provide ample mechanistic details about this relationship and compare it with the Vpx-SAMHD1 relationship. The main conclusions of this work are (1) TASOR interacts with DCAF-1 independently of Vpx (unlike SAMHD1); 2) mutants are presented, which dissect SAMHD1 versus TASOR degradation; and 3) transactivation of the viral promoter segregates with those mutants as one might expect based on HUSH degradation. This is clearly very important work and the data looks convincing. Therefore, I only have minor suggestions regarding this work.

We thank the reviewer for their comments.

The first suggestion is regarding the higher MW band that is thought to be a dimer of Vpx. It is difficult for the reviewer to judge whether this is a dimer because there are no MW markers on any the gels. Secondly, to corroborate that the dimer is indeed Vpx, I would suggest using a urea-PAGE electrophoresis setup, which most likely would reduce Vpx dimers to monomers.

- MW markers have been added on all Western-blots presented in this manuscript.

- We performed the experiment using urea-PAGE electrophoresis setup but, unfortunately, we could not reduce Vpx dimers to monomers. This non-denaturable form of Vpx has been described in Kewalramani and Emerman (Virology, 1996).

Please find here sentences extracted from this paper: “*While treatment with a reducing agent is absolutely required for detection of monomeric Vpx by SDS– PAGE, the presence or absence of reducing agent (e.g., b-me) did not significantly affect the quantity or quality of the Vpx 30-kDa species found in virions. Similar results have been obtained previously with different Vpx antisera (Kappes et al., 1993). In addition, treatment with other denaturants, such as 8 M urea, or increasing amounts of reducing agents did not appreciably decrease the level of the 30-kDa species (data not shown). Purification of the 30-kDa species from nonreducing SDS –PAGE via electroelution indicates that the majority of 30-kDa nonreduced Vpx species cannot be resolved into Vpx monomers after treatment with b-me and, thus, are not the result of a disulfide linkage. The ability of highly purified Vpx generated from bacteria to form a nondenaturable dimer strongly suggests that the 30-kDa Vpx complex detected in virions is a Vpx homodimer. In addition, Vpx translated in vitro in rabbit reticulocyte lysates also generates a 30- kDa nondenaturable complex (data not shown) … The biochemical nature of the Vpx dimerization appears to be the result of a highly stable linkage.*”

-We now show in Figure S1B that the 30-kDa Vpx species is recognized by a Vpx antibody. The signal for the 30-kDa is weak, suggesting that “dimerization” inhibits epitope recognition by the Vpx antibody.

-Below, we do demonstrate that dimerization is driven by a Vpx determinant since replacement of HIV-1 Vpr α-helix 2 by α-helix 2 from SIVsmm Vpx (which dimerizes like HIV-2 Vpx) induces apparent dimerization of HIV-1 Vpr. This experiment has not been added to the manuscript because we believe it is beyond the scope of our study (in addition, dimerization is not required for TASOR degradation).

-Nonetheless, from our experiments, we cannot totally rule out that the Vpx 30-kDa species results from a covalent attachment to a cellular protein of the same size of Vpx or from some post-translational modifications. We are now more cautious in the text. We refer to the “30-kDa Vpx form” or “apparent dimer”, instead of the dimeric form and we suggest this is a dimer.

In addition, we have changed the labels on the Figures (15 kDa-Vpx and 30-kDa Vpx instead of monomer and dimer).

-We now provide additional experiments with HIV-2 Vpx proteins (wt and mutants) tagged with a 3xFlag epitope. Flag-Vpx does not form a 30-kDa species like HA-Vpx, probably because of the size of the 3xFlag tag (23 amino acids), located in the disordered N-terminus part of Vpx that could structurally prevent the contact between two Vpx monomers.

We confirm our data with Flag-Vpx (Figures S4B, S4C and S4D).

My second suggestion is to demonstrate that TASOR degradation occurs in primary cells (lymphocytes and/or macrophages); is this something that was done in earlier work? If so I am not aware of it but it would be important.

We show in Figures 6 and S7A that Vpx-mediated TASOR degradation occurs in macrophages. Degradation in primary cells was also shown in our Chougui *et al.* manuscript Nature Microbiology, 2018.

Reviewer #1: Vpx dimerization. The authors use HIV-2 Vpx from Ghana I (line121) and found that HA-tagged WT Vpx form “non-denaturable” dimers. Intriguingly, all Vpx mutants deficient for TASOR binding (Q47A, V48A, and QV) in Figure 3D are monomers.
a) Is the difference in molecular weight between HA-Vpx wt and the mutants important for binding/function?

Our Immunoprecipitation assays show that the dimeric form of Vpx presents a stronger binding affinity to TASOR than its monomeric form. Nonetheless, we believe the difference in molecular weight (monomer versus dimer) between HA-Vpx and mutants is not important for binding and function since Vpx Q_47_A is only detected as a monomer, while being fully active (Figures 3A and 3B). In contrast, monomeric Vpx V_48_A or Vpx Q_47_A-V_48_A are inactive. Moreover, 3xFlag-tagged Vpx, which only produces a monomeric form, is still able to induce TASOR degradation and to bind TASOR and DCAF1. (Figures S4B, S4C, and S4D).

The authors should repeat the experiments with FLAG tagged Vpx wt and mutant proteins (which does not form dimers, Figure S1?)

-In agreement with our reviewer, we now provide additional experiments with a Flag tagged HIV-2 Vpx construct. Flag-Vpx does not form a 30-kDa species alike HA-Vpx. We confirm our data with Flag-Vpx (Figures S4B, S4C, and S4D).

b) How do the authors know these bands are dimers? Isn’t it possible that Vpx under these conditions is posttranslationally modified or binds to an unknown cellular cofactor?

This is, again, an interesting question/suggestion from reviewer 1, an answer to this question has been provided above.

Can the authors find similar dimers (wt) and loss of function and dimerization (mutants) in other HIV-2 or SIV Vpx proteins?

Yes, “dimerization” can also be seen with SIVsmm Vpx protein, though it is less pronounced for SIVsmm (Figure S4A). While the SIVsmm Vpx Q_47_A-V_48_A mutant is unable to reactivate HIV-1 minigenome in J-Lat A1 cells (previously shown in Chougui et al., 2018 Nature Microbiology), a decrease of binding affinity to DCAF1 and dimerization is observed for SIVsmm Vpx Q_47_A-V_48_A (Figure S4A). Nonetheless, alike HIV-2 Vpx, SIVsmm Vpx Q_47_A is only detected as a monomer and holds the same affinity binding to DCAF1 as SIVsmm Vpx WT (Figure S4A).

c) The “dimerization” of Vpx within VLPs appears to be less pronounced than in cellular lysates, especially for WT Vpx (compare S2A and S2C to Figure 3)? Why is that the case?

(of note, S2A and S2C have become S3A and S3C)

We do not know why this is the case. It is possible that formation of the “dimer” is regulated and depend for instance on the localization of the viral protein and its concentration. The localization of Vpx might be an important feature so that it dimerizes. Indeed, this hypothesis is supported by our finding that the Vpx R_70_A NLS mutant accumulates into the cytoplasm and is unable to form a dimer as much as Vpx WT, as shown below. This mutant cannot actively migrate into the nucleus, but can still be localized in the nuclear fraction likely due to passive transport (because of the small size of Vpx). These experiments have not been added, as the result does not help the general message of the manuscript (formation of the “dimer” is not important for HUSH antagonism as shown by Vpx Q_47_A).

Finally, Kewalramani and Emerman (Virology, 1996) reported the existence of the 30-kDa Vpx dimer in the virion.

Since the authors do not (cannot ?) convincingly explain the nature of the “dimer band” and given the fact that the very detailed analysis of protein-protein interactions by immunoprecipitation is the most important and major part of the manuscript, the authors need to verify the effects of the Vpx mutants on TASOR and Decaf binding in different Vpx constructs. For example, using at least a core set of FLAG-tagged Vpx mutants (Ghana) or introduce the mutations in other HIV Vpx proteins that do not form the dimers, just to rule out unwanted effects of the “dimer” band on the TASOR/DECAF interaction.

Accordingly, we now provide additional experiments with HIV-2 Vpx proteins (WT and mutants) tagged with a Flag epitope at its N-terminus part. Flag-Vpx does not form a 30-kDa species alike HA-Vpx. In a reproducible manner, we confirm our results regarding the degradation of TASOR by Flag-Vpx constructs (that do not form a dimer) (Figure S4A) and the interaction between Flag-Vpx wt and mutant proteins and DCAF1 or TASOR (Figures S4C and S4D).

Reviewer #2: (No Response)

Reviewer #3: I have suggested two small experiments in the previous section. Briefly, to demonstrate that the upper band is a dimer of Vpx, and to reproduce degradation of TASOR in primary cells.

Answer is provided in the previous section.

Reviewer #1: (No Response)

Reviewer #2: Minor issues :

In the figure legends, please specify the number of experimental repeats that were done (e.g. the immunoblots shown are representative of n experiments).

Accordingly, we have added the number of experimental repeats.

The loading control (B-actin or GAPDH as in the other immunoblots) is missing in Figure 1D.

The loading control and the IP wash control, GAPDH, have been added (Figure 1D).

As mentioned above, an interaction between TASOR and DCAF1 was reported by the authors in the absence of Vpx. The authors state that however that TASOR was not regulated by DCAF1 in the absence of Vpx, as shown by the unchanged levels of TASOR in the absence of DCAF1 (Fig S1C). However, it seems quite clear from Figure S1D and Figure 1D (assuming in the latter case that the total amount of proteins was identical between the different lines, as the loading control is missing), that TASOR seemed expressed to higher levels in the absence of DCAF1. Could the authors please comment this observation? Is this reproducible? Is it due to the ectopic expression of (a tagged version of) TASOR (i.e. HA-TASOR or TASOR-Flag)? (Does the ectopic expression change the localization of a pool of the protein for instance?...).

We thank our reviewer for highlighting this observation. Indeed, siDCAF1 treatment did not impact TASOR endogenous level in asynchronous HeLa cell culture (Fig S2B). We then conducted 2 other experiments by batch overexpressing TASOR-Flag or empty-Flag in asynchronous HeLa cells. 24h later, we divided our culture into two populations that would undergo treatment either with siCtrl or with siDCAF1 for 24h. By performing this batch transfection of TASOR-Flag and then dividing, we ensure that the basal levels of exogenous TASOR are equivalent before siCtrl and siDCAF1 treatments. Next, we performed cell fractionation by isolating, the cytoplasmic, nucleoplasmic, chromatin fractions with the help of the protocol published by Gagnon et al. 2014, Nature protocols. As presented below, DCAF1 inhibition does not upregulate the endogenously (in agreement with Fig S2B) or exogenously expressed TASOR protein and does not alter their respective distribution. We apologize that the results presented in Figures 1D and S2C seem to suggest a role of DCAF1 in TASOR protein turnover in asynchronous HeLa cells. The experiment setup (siRNA treatment then expression of TASOR-Flag) cannot give any information on the role of DCAF1 since the basal expression levels of exogenous TASOR would be different between conditions. However, it might be possible that this event could occur in a specific spatio-temporal window, for example at a specific time point of the cell cycle or after DNA damage at a specific genome locus. The hypothesis raised by our reviewer is actually a very interesting point and requires further investigations. Finally, this experiment setup does not contradict the fact that Vpx can interact with TASOR independently from DCAF1. We have modified the paragraph in our discusion:

*“*We showed that DCAF1 does not regulate endogenous TASOR levels in asynchronized cells (S2B Fig). However, it could be that DCAF1 controls TASOR turnover in a specific cellular context, for instance in a specific window along cell cycle progression or upon DNA damage. Further investigations are required to determine whether DCAF1 could serve as an ubiquitin ligase adaptor for TASOR degradation in physiological conditions*.”*

Notes: GAPDH, ZFC3H1 and Histone H3 show the purity of the Cytoplasmic, Nucleoplasmic and Chromatin fractions respectively.

Fig. 3D: there seemed to be less DCAF1 co-immunoprecipitated with TASOR-Flag in the presence of Vpx QV mutant compared to the condition where Vpx is absent: is it reproducible and significant?

In Figure 3D, there is less DCAF1 co-immunoprecipitated with the HA-Vpx-QV mutant. This is reproducible and this is now confirmed with the Flag-Vpx-Q_47_AV_48_A mutant (Figure S4C). Next, when we express Vpx WT or Q_47_A mutant for example, that are not affected for DCAF1 binding, we detect a stronger affinity of TASOR-Flag for DCAF1 (Figures 2D,3E, S6B). So, Vpx binding to TASOR would indirectly strengthen the affinity of TASOR for DCAF1. In contrast, Vpx mutants as V_48_A, or Q_47_AV_48_A that are less affine for DCAF1 (Figure 3D) would not reinforce the affinity of TASOR for DCAF1, so we do not detect a stronger signal for the DCAF1 band when immunoprecipitating TASOR-Flag (Figure 3E).

We agree that in this Figure 3E (we believe the reviewer refers to 3E), we seem to see a weaker signal for DCAF1 co-immunoprecipitated with TASOR-Flag in the Q_47_AV_48_A condition compared to the condition where Vpx is absent, but we cannot conclude that the difference is significant and then reproducible after several repeats.

p11 “… macrophages, in which both TASOR and SAMHD1 are present”: either add restriction (SAMHD1 restriction) or replace “present” by “active”, as SAMHD1 is ubiquitously expressed but active in myeloid cells

This has been done.

Reviewer #3: N/A
